# Lactylation: A Novel Epigenetic Regulator of Cellular Senescence

**DOI:** 10.14336/AD.2025.0277

**Published:** 2025-03-23

**Authors:** Caiyu Sun, Jiaxuan Li, Lei Dong, Yakui Mou, Bei Zhang, Xicheng Song

**Affiliations:** ^1^Department of Otorhinolaryngology, mHead and Neck Surgery, Yantai Yuhuangding Hospital, Qingdao University, Yantai, Shandong, China.; ^2^Department of Immunology, School of Basic Medicine, Qingdao University, Qingdao, Shandong, China.; ^3^Shandong Provincial Key Laboratory of Neuroimmune Interaction and Regulation, Yantai Yuhuangding Hospital, Yantai, Shandong, China.; ^4^Shandong Provincial Clinical Research Center for Otorhinolaryngologic Diseases, Yantai Yuhuangding Hospital, Yantai, Shandong, China.; ^5^Yantai Key Laboratory of Otorhinolaryngologic Diseases, Yantai Yuhuangding Hospital, Yantai, China.

**Keywords:** lactylation, cellular senescence, metabolic reprogramming, post-translational modification, histones

## Abstract

Cellular senescence is the basic unit of organismal aging, a complicated biological process involving several cell types and tissues. It is also an important mechanism by which the body responds to damage and potential carcinogenesis. However, excessive or abnormal cellular senescence can lead to tissue functional degradation and the occurrence of diseases. In recent years, the role of epigenetic modifications in cellular senescence has received extensive attention. Lactylation, a novel post-translational modification derived from lactate, has recently gained significant attention as a key factor in cellular metabolism and epigenetic regulation, gradually demonstrating its importance in the regulation of cellular senescence. This review emphasizes the bidirectional causal relationship between lactylation and cellular senescence, highlighting its potential as a therapeutic target for aging-related diseases.

## 1. Introduction

Cellular senescence is induced by diverse factors, including organelle stress, DNA damage, telomere dysfunction and oncogene activation. This process is related to tumor development, tissue repair, embryogenesis, and biological aging [1-3]. Conventional wisdom has long posited that the process of cellular senescence engenders exclusively deleterious effects, including the deterioration of tissue function and the promotion of the development and progression of chronic diseases. However, recent studies have demonstrated that cellular senescence exhibits dual characteristics, functioning in a manner that is both detrimental and beneficial. Specifically, the process of cellular senescence has been observed to play a proactive role in the developmental process of embryos and the healing process of wounds. Moreover, the induction of tumor cellular senescence has emerged as a promising therapeutic approach [4-7]. Cellular senescence constitutes the foundational principle of the aging process and a multitude of diseases. With an ageing global population, it is imperative to investigate the mechanisms underlying cellular senescence, identify strategies to postpone aging, and develop therapeutic targets for associated diseases [8-11].

Lactate is a key metabolite generated through the Warburg effect, serving dual roles as an energy source and metabolic byproduct. Recently, its role in histone lactylation has received considerable attention [12, 13]. In 2019, the research team led by Professor Zhao from the University of Chicago first discovered that lactate could serve as a precursor for for lactylation of histone lysine. They also confirmed that the lactylation of histones could directly promote the gene transcription of chromatin. This significant accomplishment marked the beginning of lactylation study [14]. Subsequent studies have demonstrated that lactylation is associated with cell signal transduction and transcriptional regulation, and critically involved in the development of numerous diseases, including tumors, cardiovascular diseases, and neurodegenerative disorders [15-18]. Notably, in cellular responses to environmental or metabolic demands changes, lactylation modulates the expression of metabolism-related genes, thus maintaining intracellular environment stability [19-21].

While research findings on cellular senescence and lactylation in their respective domains have proven fruitful, the direct correlation between them remains in nascent stages. A more exhaustive examination of the mechanisms underlying the role of lactylation in cellular senescence will not only bridge this knowledge gap but also provide innovative theoretical underpinnings and hitherto unexplored therapeutic targets for mitigating and addressing age-related pathologies. These advances are of immense value for enhancing human health and confronting the challenges posed by an aging population.

## 2. Lactylation

### 2.1 Lactate and Lactylation

With the development of life science, lactate is not only considered as metabolic waste, but also as an important metabolite in many physiological processes [22-25]. Histone lactylation was first identified and characterized in 2019 using high-performance liquid chromatography-tandem mass spectrometry (HPLC-MS/MS). Since then, lactate has been identified as a key mediator of epigenetic regulation [14]. Lactylation is a post-translational modification of protein, in which the lactyl moiety transfers from a donor to specific amino acid residues (such as lysine) on proteins. This process primarily occurs in histones (similar to histone acetylation), thus activating or inhibiting the expression of related genes [26, 27]. Histone lactylation is predominantly the result of the modification of lysine residues on histones by L-lactic acid. However, lysine lactylation (Kla) does not exist independently, and there are two isomers with similar structures: D-lactyl-lysine and N-ε-(carboxyethyl)-lysine. In 2024, the research team led by Professor Zhao successfully distinguished these three modified isomers through analytical chemistry and mass spectrometry methods. The different modifications show significant differences in substrate sources, target proteins and modification processes [28]. D-lactyl-lysine primarily exerts its effects on non-histones, particularly glycolytic enzymes, and regulates glycolysis through a negative feedback mechanism [29]. Professor Cao's team recently discovered that the glycolytic branch metabolite S-D-Lactoylglutathione (SLG) can directly induce the D-lactylation of protein and transfer the lactate group to lysine through a cysteine intermediate. This non-enzymatic lactylation process has been shown to significantly inhibit the inflammatory response in immune cells [30]. The modification of lactate was demonstrated at a later stage, and although numerous studies related to lactate did not completely extend to the lactylation of key molecules in cells, they can serve as a reminder of the potential effects of lactylation.

### 2.2 Key Factors Regulating Lactylation

In contrast to acetylation, lactylation is contingent on the intracellular concentration of lactate and the activity of lactate dehydrogenase (LDH). Its regulation is influenced by the metabolic microenvironment, which can be influenced by factors such as hypoxia or elevated glucose levels [31-34]. Furthermore, the writers or erasers of lactylation determine the reaction of enzymatic lactoylation. Among them, histone acetyltransferase CREB-binding protein (CBP) and its closely related p300 protein (CBP/p300) or the lysine acetyltransferase (KAT) are common "writers", while members of the deacetylase family such as sirtuins or HDAC are common "erasers" [14, 21, 27]. In addition to the similar modifying enzymes, both lactylation and acetylation can modify histones and non-histone proteins, thereby regulating gene transcription and protein functions and influencing the life activities of cells. However, whether lactylation can regulate or compete with acetylation remains to be studied [21, 35]. The results of global acylome technology for lactylation and acetylation show that the two have different preferences for amino acids surrounding lysine residues. Acetylation prefers hydrophobic amino acids Y and F at the +1 position, while lactylation shows a preference for amino acids such as K, G, and P [36]. It can be seen that lactylation and acetylation are quite similar, which adds difficulties to the research and targeting of lactylation. Lactylation also has regulatory effects on other modifications. For example, research shows that histone lactylation can promote m6A methylation [22, 37, 38] and can also drive m1A demethylation [23].

## 3. Lactylation and Cellular senescence

### 3.1 Cellular Senescence

Cellular senescence is a process in which the proliferation, differentiation and physiological functions of cells gradually decline over the course of life. It is a stable terminal growth arrest state in which cells are unable to proliferate even under optimal growth conditions and mitotic stimulation [1]. The hallmarks of cell senescence are crucial for understanding the relevant biological mechanisms and are of great significance for both normal cellular senescence and age-related diseases. Cellular senescence is typified by a series of highly distinctive and notable hallmarks. Morphologically, the nucleus undergoes hypertrophy, with the nuclear membrane exhibiting folding; the cytoplasmic bridges and lysosomes are increased, and lipofuscin is accumulated. In terms of physiological features, these principally comprise cell cycle arrest, senescence-associated secretory phenotype (SASP), DNA damage, alterations in nuclear architecture, modifications to cell surface markers, abnormal telomere shortening or dysregulated telomere homeostasis, mitochondrial dysfunction, upregulation of anti-apoptotic signaling pathways, and metabolic reprogramming [1, 8, 10, 39, 40]. Among them, the upregulation of anti-apoptotic signaling pathways such as B-cell lymphoma-2 (BCL2) and BCL-2-associated X protein (BAX) during cellular senescence seems to oppose apoptosis. However, it supports apoptosis as the ultimate mechanism for cell cycle exit [41]. Apoptosis is a crucial mode of cell death, which refers to the genetically regulated, orderly cells death to maintain the stability of the internal environment. It has two pathways: intrinsic and extrinsic. Mitochondria are crucial in the intrinsic pathway for responding to internal stimuli. This pathway is regulated by pro-apoptotic members of the BCL-2 family (such as BAK and BAX), with signal transduction occurring through caspase - 9. The convergence of these two pathways takes place upon the activation of caspase-3 and caspase-7. Once activated, these caspases execute proteolytic cleavage events, leading to the subsequent activation of downstream targets [42, 43]. Many stimuli that induce senescent phenotypes can also trigger apoptosis, such as DNA damage and cell cycle arrest [44]. However, the molecular mechanism that further determines the choice between apoptosis and senescence remains unclear. The fate of cells may depend on the intensity and duration of the initial stimulation, along with the type and cell type of injury [45-47].

### 3.2 Cellular Senescence and Diseases

Cellular senescence pervades the entire life cycle of organisms and represents the inevitable destination of cellular life activities [48]. In the body, cellular senescence and the renewal of new cells always maintain a dynamic equilibrium. However, cellular senescence is also considered a driving factor for aging and age-related diseases [49]. A large number of examples of cellular senescence at the sites of aging pathology have been reported. Nevertheless, it remains unclear whether these phenomena of cellular senescence are the causes of diseases or the consequences of pathological changes [50]. Age-related diseases mainly occur in the tissues which have already become dysfunctional due to the aging process with the accumulation of senescent cells, such as atherosclerosis, osteoarthritis, Alzheimer's disease, etc. [51-53]. Although cancer is not a typical aging-related disease, cellular senescence plays a crucial role in different stages of tumor development and evasion [54]. The activation of oncogenes can trigger cellular senescence. This process, which is designated as oncogene-induced senescence (OIS), is capable of exerting an inhibitory effect on tumor growth [55]. Therefore, cellular senescence is a physiological tumor suppression mechanism that can inhibit the progression from benign tumor lesions to malignant tumors. However, in some cases, senescent malignant and non-malignant cells can acquire tumor-promoting characteristics [56]. Senescent tumor cells can also shape the tumor microenvironment through SASP, either promoting or inhibiting tumor progression at different stages of progression [54, 57]. Meanwhile, senescent cells can also induce the repair and regeneration of tissues through SASP, thus establishing a balance between senescent cells and newly generated cells [58, 59]. Therefore, the research on the regulatory mechanisms of senescent cells is of great significance for aging-related diseases, injury repair, tumor treatment, and other aspects.

## 3.3 Regulation of Lactylation on Cellular Senescence

### 3.3.1 Regulation of Lactylation on SASP

**Figure 1.**
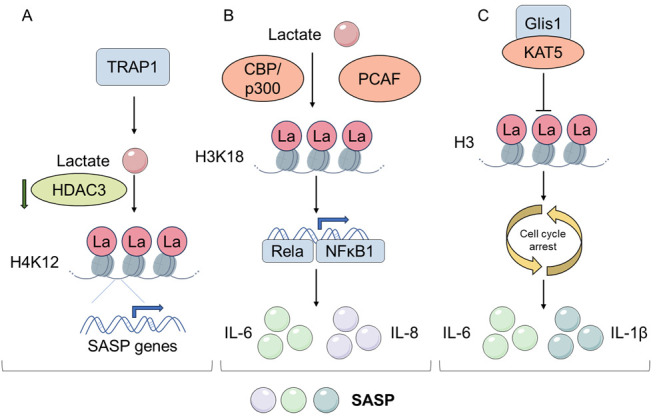
**Lactylation can promote the secretion of SASP**. (**A**) TRAP1 significantly enhanced aerobic glycolysis, thereby inducing lactate accumulation. Lactate increases the H4K12la by downregulating HDAC3. H4K12la enriches in the promoter regions of SASP, activating SASP transcription and exacerbating the senescence of VSMC. (**B**) Lactate accumulation induces H3K18la in BV2 cells, which increases the binding to the promoters of Rela and NFκB1, directly stimulating the NFκB signaling pathway and thus upregulating IL-6 and IL-8. And the acetyltransferases p300/CBP and PCAF are histone lactylases in cells. (**C**) Glis1 can interact with KAT5, reducing the level of histone lactylation, downregulating the levels of p16 and p21 and slowing down the cell cycle. Compared with STZ-induced diabetic kidney disease DKD mice, the Glis1 overexpression mouse model reduces the levels of IL-6 and IL-1β.

Senescent cells can secrete a large number of chemokines, cytokines, growth factors and proteases called SASP, mainly including interleukin -6(IL-6) and tumor necrosis factor -α(TNF-α), and so on. Lactylation can directly or indirectly regulate the SASP, affect cellular senescence, and thus participate in the inflammatory response, tissue repair, and tumor occurrence and development (Fig. 1) [60-63]. Lactate is a biological factor derived from the tumor microenvironment that can accurately reflect the development of SASP in patients. Furthermore, it can be used to evaluate the amplitude of SASP in patients following cancer treatment [64]. Senescence of vascular smooth muscle cells (VSMC) critically contributes to the progression of atherosclerosis. The research indicates that tumor necrosis factor receptor-related protein 1 (TRAP1) can induce lactate accumulation in senescent VSMC, which increases histone H4 lysine 12 lactylation (H4K12la) by blocking the histone delactylating enzyme, HDAC3. H4K12la is enriched in the promoter of SASP, which activates the transcription of SASP, exacerbates senescence of VSMC, and promotes atherosclerosis [65]. It was also found that histone H3 lysine 18 lactylation (H3K18la) and pan-lysine lactylation (pan-Kla) were significantly upregulated in aging microglia and the hippocampi of naturally aging mice and AD mouse models. The increased H3K18la directly stimulated the NFκB pathway by increasing binding to Rela and NFκB1 promoters, thereby upregulating IL-6 and IL-8. Meanwhile, the study confirmed that the acetyltransferases p300/CBP and PCAF are histone lactyltransferases in cells such as 293T, Hela and BV2 [66].

Research demonstrated that senescence is accompanied by chronic, aseptic, and mild inflammation, which trigger aging-related diseases. These chronic diseases are not solely outcomes of senescence and inflammatory senescence, and they also exacerbate the senescence process [67]. Recent studies have revealed that compared with peripheral blood mononuclear cells (PBMCs) of young adults, PBMCs of the elderly exhibit lower levels of S-D-lactoglutathione (SLG) and weaker protein lactylation. This discrepancy suggests that the glyoxalase 2 (GLO2)/SLG feedback loop is weakened in the elderly, possibly contributing to mild chronic inflammation, also referred to as chronic SASP. This process is caused by the non-enzymatic direct induction of D-lactylation of proteins during the activation of innate immunity. Lactylation is preferentially enriched in proteins involved in immune activation and the inflammatory pathway. The D-lactylation of K310 of the RelA protein can attenuate inflammatory signal transduction and transcription activity of NF-κB, thereby contributing to the restoration of immune homeostasis [30]. Of course, the initial investigation regarding lactylation pertained to its modulation of inflammation. Research revealed the existence of an endogenous "lactate clock" within macrophages. Lactate and histone lactylation exert a positive influence in driving the expression of M2-like genes during the polarization of M1 macrophages [14]. Subsequently, other studies have delved into and complemented the mechanisms underlying the regulation of macrophage polarization and function by lactylation. These studies indicate that lactylation facilitates the transition of cells from a pro-inflammatory phenotype to an anti-inflammatory one [68-71]. Moreover, certain studies have indicated that lactylation is capable of promoting the inflammatory response [72-74]. Additionally, research has shown that lactylation can regulate the inflammatory processes involving other cells, such as monocytes and Th17 cells [20, 24]. Consequently, lactylation mainly plays a role in regulating homeostasis in the modulation of inflammation.

### 3.3.2 Regulation of Lactylation on Cell Cycle

Lactate has a significant impact on the cell cycle and can be used as an indicator. Liu et al. found that in the G1 phase of HeLa cells, glucose metabolism was dominated by the tricarboxylic acid (TCA) cycle, whereas in the S phase, it was more inclined to glycolysis. Furthermore, isocitrate dehydrogenase (IDH)1/2, an important TCA enzyme, is coupled to the cell cycle and metabolism. And S-phase kinase-related protein 2 (SKP2), a cell cycle regulator, regulates the stability of IDH1/2. SKP2 inhibition can upregulate IDH1 expression and shifts the metabolism of cancer cells from glycolysis to the TCA cycle or serves as a feasible approach to suppress tumor cell proliferation. Therefore, the lactate-generated acidic microenvironment significantly promotes cancer cell proliferation via cell cycle regulation [75]. Lactate accumulates in rapidly dividing cells because of the need to improve glucose catabolism to support cell proliferation. Lactate forms a complex with zinc at the active site of SUMO Specific Peptidase 1 (SENP1), thereby inhibiting SENP1 and stabilizing the sumoylation of the two residues on APC4, thereby promoting the combination of Ubiquitin-conjugating enzyme E2 C (UBE2C) and anaphase promoting complex (APC/C). The direct regulation of APC/C by lactate stimulates the periodic degradation of cyclin, which facilitates efficient exit from mitosis in human cells [76].

Some studies have directly confirmed that non-histone lactylation in cells can regulate the expression of cyclins, thus influencing cell cycle changes. Research shows that lactylation at lysine 348 of cyclin E2 (CCNE2 K348la) can promote the growth of hepatocellular carcinoma (HCC) cells. Furthermore, the activation of SIRT3 by honokiol induces the apoptosis in HCC cells by erasing the CCNE2 K348la, thereby inhibiting the growth of HCC cells in vivo [16]. Centromere proteins (CENPs) are key mitotic protein complexes that are involved in centromere assembly and chromosomal segregation. CENPA is significantly upregulated in HCC, and high CENPA expression is associated with poor prognosis in patients with HCC. CENPA can be lactylated at the lysine 124 (K124), which enhances the expression of its target genes. CENPA-YY1-Cyclin D1 (CCND1)/Neurocilin 2 (NRP2) axis can promote cancer [77]. The regulation of the cell cycle by lactylation may contribute to the rapid proliferation.

Cell cycle arrest in senescent cells is mainly initiated by the p16INK4a/Rb and p53/p21CIP1 tumor suppressor gene pathways [78, 79]. Among them, p53 is activated under stress such as DNA damage, and p21 gene expression is consequently activated. P21 can inhibit the activity of cyclin-dependent kinase (CDK), resulting in cell cycle arrest and cell arrest in the G1 or G2/M phase. Similarly, upregulation of the p16INK4a will inhibit the activity of CDK4/6 and put cells into a state of senescence [80, 81]. In diabetic nephropathy, researchers found that GLIS family zinc finger 1 (Glis1) can bind and interact with lactoyltransferase KAT5, reducing the interaction of histone and KAT5, decreasing the lactylation level of histone, and alleviating the senescence of renal tubular epithelial cells. When the level of Glis1 decreases, the SA-β-Gal activity in the cytoplasm is significantly enhanced, and the expression levels of p16, p21, and decoy receptor 2 (DcR2) increase. Similarly, the phenomena are detected in renal tubular epithelial cells (RTECs) of streptozotocin (STZ)-induced diabetic kidney disease (DKD) mice. Additionally, compared with STZ-induced DKD mice, Glis1 overexpression decreased the levels of IL-6 and IL-1β (Fig. 1C) [82]. Some studies have also found that, as a transcription factor, Glis1 can enhance glycolysis and increase the levels of acetyl coenzyme A and lactate in cells by binding and regulating glycolytic genes, thereby increasing the acetylation (H3K27Ac) and lactylation (H3K18la) of pluripotent gene loci, reprogramming senescent cells into pluripotent cells, and improving genome stability [83].

Meanwhile, studies have shown that p53 can directly affect the lactylation process. Bone morphogenetic protein (BMP) is increased by continuous activation of ACVR1 in cranial nerve crest cells, which inhibits glycolytic activity and blocks lactate production through a p53-dependent process, resulting in severe midline facial defects [84]. Lactylation of p53 can regulate and influence the progression of various diseases. Researchers found that alanyl-tRNA synthetase 1 (AARS1) can bind to lactate, catalyze the formation of lactate-adenosine monophosphate (lactate-AMP), and then transfer lactate to lysine 120 and lysine 139 in the DNA binding domain of p53, which hinders its liquid-liquid separation and transcriptional activation, and promotes tumor progression. However, beta-alanine can disrupt the combination of lactate and AARS1, reduce p53 lacylation, and reduce tumorigenesis in animal models [85]. In the treatment of advanced prostate cancer (PCa), researchers found that SLC4A4 can mediate the lactylation of p53 through the NF-κB/STAT3/SLC4A4 axis, which eventually leads to the development of enzalutamide resistance and the progression of PCa [86]. However, in microglia cells, the lysine lactylation modification of p53 contributes to the pro - inflammatory activation of LPS-induced BV2 cells through NF-κB pathway under hypoxia, and inhibition of lactate production can alleviate neuroinflammatory injury [87]. Therefore, whether p53 mainly regulates cell cycle-related proteins such as p21 or not, lactylated p53 may regulate cellular senescence. However, during tumor progression, as the level of lactylation rises, the TP53 protein undergoes degradation, which may delay the further senescence of the tumor. In ocular melanoma, an increase in histone lactylation levels, especially an increase in H3K18la, induces the transcription of YTHDF2, and YTHDF2 then recognizes m6A-modified PER1 and TP53 mRNA and promotes their degradation [88]. Recent studies have shown that during the development of HCC, local hypoxia drives the evolution of cancer cell subsets into a cell lineage with high lactate dehydrogenase A (LDHA) expression. This results in intracellular lactate accumulation, which in turn activates the expression of the NDRG1 gene through histone lactylation. Conversely, NDRG1 resists stress-induced cellular senescence by regulating the glycogen synthase kinase-3β (GSK-3β)-p53 signaling pathway [89].

### 3.3.3 Lactylation and Mitochondrial Dysfunction

With the progressive senescence of cells, the structure and function of mitochondria deteriorate, manifesting as a decline in mitochondrial membrane potential, a decrease in respiratory chain complex activity, and an escalation in reactive oxygen species (ROS) production. The progression of mitochondrial dysfunction, exacerbates cellular senescence, thereby establishing a self-perpetuating cycle [90]. Lactate, a byproduct of cellular metabolism, redirects glucose metabolism to the pentose phosphate pathway, elevating NADPH levels. This shift in metabolic flux activates the PI3K/Akt pathway and enhances NADPH oxidase 4 (NOX4). Notably, lactate promotes the production of ROS and cartilage injury. Furthermore, it enhances the expression of catabolic enzymes, diminishes the synthesis of type II collagen, stimulates the expression of inflammatory cytokines, and induces cartilage hypertrophy and aging [91]. Tumor research has also demonstrated that PDK4-mediated lactate production enhances the activation of NOX1, thereby effectively driving the production of ROS and promoting the development of SASP. This process was accompanied by an increase in DNA damage response (DDR) signal transduction. Inhibition of PDK4 activity or the lactate production pathway has been shown to inhibit aging-related phenotypes, especially the SASP [64].L-lactate augments cytoprotective responses by inducing a mild burst of ROS, including activation of the unfolded protein response (UPR) and nuclear factor erythroid 2-related factor 2 (NRF2). This triggers the antioxidant defense and survival promotion pathways (PI3K/AKT and endoplasmic reticulum (ER) partners). These findings suggest that L-lactate may serve as a target for cell protection and treatment of aging-related diseases [92]. Research has demonstrated that L-lactate can safeguard skin fibroblasts from dysfunction associated with mitochondrial aging through a process referred to as mitotic stimulation [93]. Concurrently, extracellular lactate can induce Th17 cells to secrete IL-2, which is driven by ROS, and results in a substantial reduction in IL-17A production and upregulation of forkhead box P3 (*Foxp3*) expression. During this process, whole-genome H3K18la level increases, which induces the upregulation of NF-κB and mitogen-activated protein kinase (MAPK) signaling pathways. Moreover, H3K18la is enriched in the proximal promoter region of *Foxp3* [20]. This study did not directly clarify the relationship between ROS and H3K18la, only observing the co-existence of the phenomena in T cells. Based on the above studies, lactate can directly or indirectly promote the production of ROS, thereby influencing the life activities of cells. However, studies have shown that histone lactylation can also directly affect and regulate the production of ROS. Notably, histone lactylation can directly affect and regulate the production of ROS. Further research suggests that the presence of H3K18la modulates the process of transcriptional activation of the *duox* gene, consequently inducing the generation of ROS. Consequently, ROS generation promotes the H3K18la, thereby establishing a positive feedback loop [94]. Studies have shown that the lactylation modification of mitochondrial proteins can directly impact mitochondrial function. In sepsis-induced acute kidney injury (SAKI), down-regulation of SIRT3 mediates hyperacetylation and inactivation of pyruvate dehydrogenase E1 subunit alpha 1 (PDHA1), leading to excessive lactate production. Lactate can mediate the lactylation of the lysine 20 (K20la) of mitochondria mitogen 1 (Fis1), promoting excessive mitochondrial fission, which in turn leads to adenosine triphosphate (ATP) depletion, excessive production of mitochondrial ROS and apoptosis. The use of sodium dichloroacetate (DCA) to activate PDHA1 or the overexpression of SIRT3 can reduce the lactate levels and Fis1 K20la, thereby alleviating SAKI [95]. During the process mediated by the above mentioned Fis1 K20la, there is a significant up regulation of BCL2 and down regulation of BAX. This is contrary to the currently known process of cellular senescence, as the apoptosis-related markers usually show a downward trend during senescence [96, 97]. However, the ultimate fate of senescent cells still seems to need further exploration [41, 98]. Although evidence shows that lactylation can regulate the production of ROS, there is currently no direct evidence suggesting that lactylation affects cellular senescence by regulating mitochondrial ROS.

**Figure 2.**
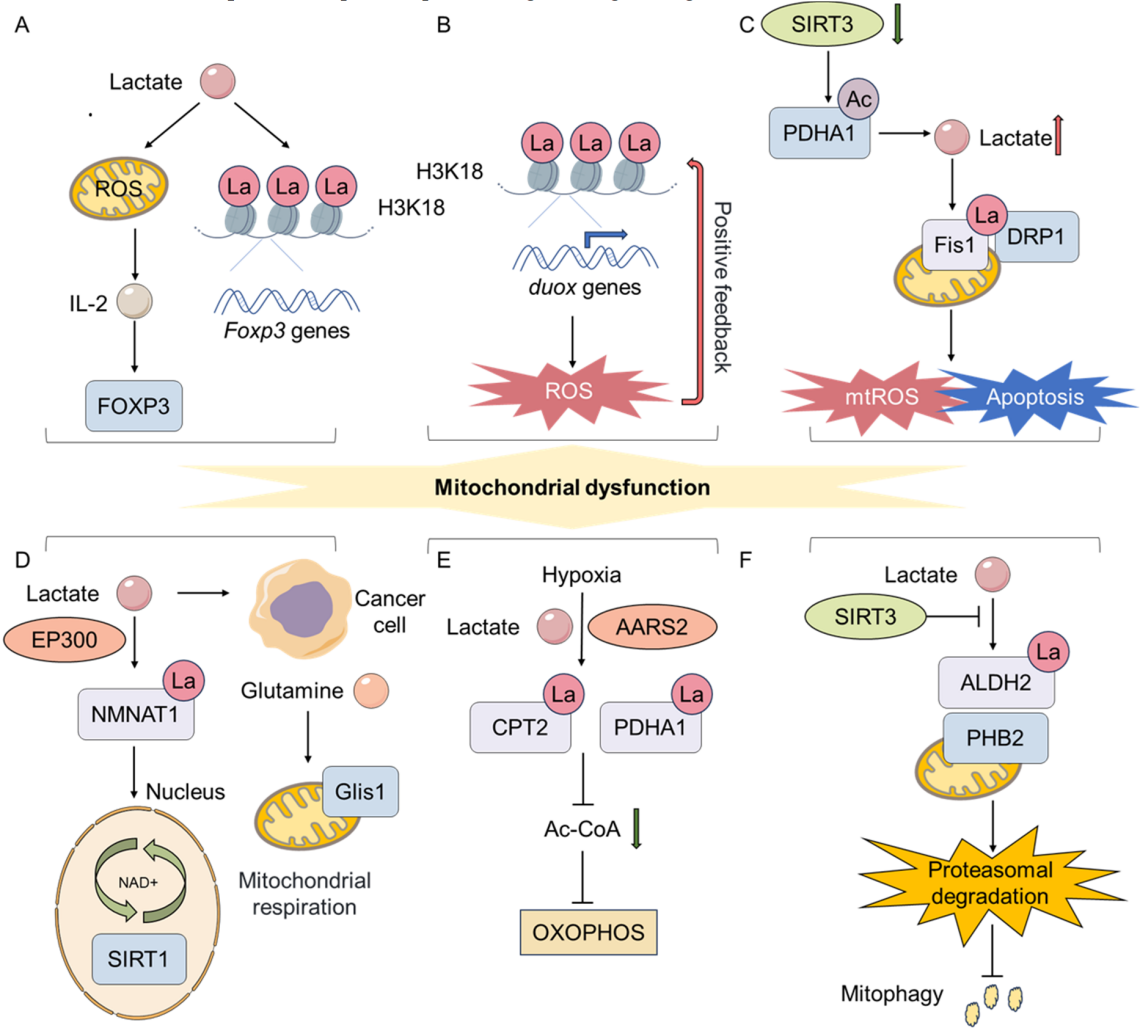
**Lactylation can regulate the mitochondrial function of cells**. (**A**) Lactate can induce Th17 cells to significantly reduce their production of IL-17A and upregulate Foxp3 expression through ROS-driven IL-2 secretion. And H3K18la is enriched in the proximal promoter region of Foxp3. (**B**) H3K18la regulates the transcriptional activation of the duox gene, leading to the production of ROS. And ROS can also promote H3K18 lactylation, forming a positive feedback loop. (**C**) Downregulation of SIRT3 mediates hyperacetylation and inactivation of PDHA1, resulting in excessive lactate production. Lactate can mediate Fis1 K20la, which in turn leads to overproduction of mtROS and mitochondrial apoptosis. (**D**) Lactate can enhance the NMNAT1-mediated NAD+ salvage synthesis pathway, and NMNAT1 can be lactylated at K128la by EP300. The inhibitory effect of lactate on cell death under glucose deprivation mainly depends on the mitochondrial respiration supported by GLS1-mediated glutaminolysis. (**E**) Hypoxia causes the accumulation of AARS2. And, by lactylating CPT2 and PDHA1, it enables the influx of acetyl-CoA and inhibits OXPHOS. (**F**) ALDH2 can be lactylated at K52la, promoting the ubiquitination-proteasomal degradation of PHB2, thus inhibiting and exacerbating mitochondrial dysfunction. Upregulating SIRT3 can reduce the lactylation of ALDH2.

Research has demonstrated that L-lactate primarily depends on glutamine catabolism to support mitochondrial respiration and promote the survival of pancreatic cancer (PAAD) cells under glucose deprivation. In this process, L-lactate augments the NMNAT1-mediated nicotinamide adenine dinucleotide (NAD+) salvage synthesis pathway, inactivate the p-38 MAPK pathway, and inhibit the transcription of DNA damage-inducible transcript 3 (DDIT3). The lysine 128 of NMNAT1 has been identified as the primary site of lactylation, and EP300 has been determined to be the pivotal enzyme that catalyzes this process. The study demonstrates that lactylation of NMNAT1 enhances its nuclear localization and enzyme activity, and promotes tumor growth, which depends on the activity of Sirt1[99]. Oxidative phosphorylation (OXPHOS) consumes oxygen to generate ATP. Research shows that mitochondrial protein lactylation can integrate intracellular hypoxia and lactate signals to regulate OXPHOS. Hypoxia leads to the accumulation of the lactyltransferase mitochondrial alanyl-tRNA synthetase 2 (AARS2). By lactylating CPT2 and PDHA1, it restricts the influx of acetyl-CoA produced from pyruvate and fatty acid oxidation, inhibiting OXPHOS. This process can be reversed by SIRT3. The above-mentioned phenomenon has been demonstrated in mouse muscle cells. Exercise-induced intracellular hypoxia triggers lactylation, which restricts the fatigue onset time during high-intensity endurance running. Moreover, this fatigue onset time increases or decreases in tandem with the changes in the lactylation level [34]. In the kidneys of patients with acute kidney injury (AKI) and in murine models, the lactate levels and pan-Kla levels increased significantly. Moreover, the lactylation activity in the injured proximal tubular cells increased markedly. During the process, aldehyde dehydrogenase 2 (ALDH2) can be lactylated at lysine 52 (K52la), which promotes the ubiquitination-proteasome degradation of mitochondrial autophagy receptor prohibitin 2 (PHB2) and exacerbates mitochondrial dysfunction. Conversely, SIRT3 up-regulation reduces the lactylation level of ALDH2, thereby alleviating renal tubular injury and mitochondrial dysfunction [100]. SIRT3 is one of the members of the sirtuins family. As an essential mitochondrial metabolic regulatory enzyme, SIRT3 plays a crucial role in mitochondrial homeostasis and can influence cellular senescence [101, 102]. Sirtuins are a class of NAD+-dependent deacetylases that are highly conserved. Their functions within cells vary depending on their locations. Research indicates that the activity of sirtuins diminishes with age, and as key eraser of lactylation, this suggests that lactylation may gradually increase during the cellular senescence process [103-107]. Studies also suggest that the absence of any sirtuin accelerates the senescence of human stem cells, which also supports this view [108].

Lactate pre-treatment reduced the production of ROS and the disruption of mitochondrial membrane potential induced by H_2_O_2_ in ARPE-19 cells through the activation of autophagy, thus playing a protective role against oxidative stress-induced retinal degeneration [109]. However, the study also mentioned that lactate treatment could induce a mild burst of intracellular ROS levels and trigger autophagy [110]. Moreover, research shows that lactate can link glycolysis with autophagy through lactylation. Under serum deprivation conditions, UNC-51-like kinase 1 (ULK1) phosphorylates LDHA, increasing its enzymatic activity and promoting lactate production. Lactate further promotes the lipid kinase activity of vesicular protein sorting 34 (Vps34) through lactylation mediated by the acyltransferase KAT5/TIP60 and promotes autophagic flux and lysosomal transport [111]. Tumor-derived lactate promotes the transcription of RUBCNL/Pacer through H3K18la and promotes the maturation of autophagosomes by interacting with BECN1 (beclin 1), thus promoting the resistance of colorectal cancer to bevacizumab [112]. The relationship between autophagy and senescence is controversial. Autophagy may induce senescence, or it may inhibit senescence [113-116]. In summary, lactylation can influence cellular processes by regulating ROS production, mitochondrial apoptosis, mitochondrial respiration, and autophagy (Fig. 2). However, the further impact of these effects on cellular senescence remains to be explored.

### 3.3.4 Lactylation and DNA Damage

**Figure 3.**
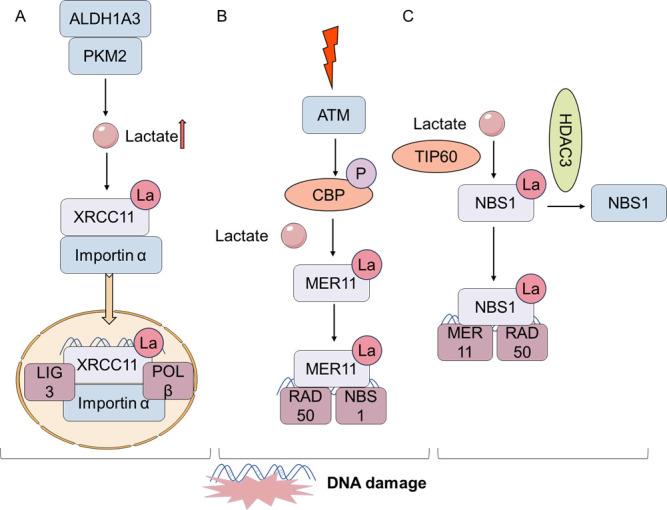
**Lactylation can promote DDR**. (**A**) ALDH1A3 can interact with PKM2, promoting lactate accumulation and inducing XRCC1 K247la. And lactylated XRCC1 has a stronger affinity for importin α and can be transported into the nucleus more, enhancing DNA repair. (**B**) MRE11 is lactylated at K673 by the CBP in response to DNA damage and is dependent on CBP phosphorylation. (**C**) TIP60 can lactylate NBS1 K388la, which is important for the formation of the MRN complex (MRE11/RAD50/NBS1) and DNA HR.

Prolonged activation of DDR induces senescence [117-119]. It has been demonstrated that ALDH1A3 can interact with PKM2, thereby enhancing its tetramerization, promoting the accumulation of lactate in glioblastoma stem cells (GSCs), and inducing the lactylation at the lysine 247 site of XRCC1. The lactylated form of XRCC1 exhibited a heightened binding affinity for the importin α, facilitating its enhanced transport to the nucleus and, consequently, potentiating DNA repair processes [120]. Lactylation stimulates DNA repair processes involving homologous recombination repair (HR) in tumor cells. MRE11 is an important HR protein that undergoes lactylation at K673 by CBP in response to DNA damage and dependents on ATM phosphorylation by CBP. The lactate-induced combination of MRE11 and DNA facilitates DNA terminal excision and HR. Conversely, elevated lactate levels within cancer cells have been shown to induce resistance to chemotherapy, particularly through the inactivation of MRE11, a process that can be counteracted by targeting lactate accumulation [121]. It has been proposed that TIP60 can function as a lysine lactate transferase under conditions of lactate accumulation, thereby facilitating the formation of the MRN complex (MRE11/RAD50/NBS1) and the HR of DNA through the lactylation of NBS1 K388 [122]. Research shows that lactylation can significantly induce an enhancement the DDR ability, and this seems to delay further cellular senescence (Fig. 3).

### 3.3.5 Lactylation and Cell Telomere

Telomere function-related regulation is one of the earliest and most common mechanisms of cellular senescence induction [123]. It has been demonstrated that LKB1 can induce the senescence of lung adenocarcinoma cells by inhibiting telomerase activity and inducing telomere dysfunction by regulating telomerase reverse transcriptase (TERT) expression. LKB1 has been observed to reduce lactate production and inhibit the lactylation of histones H4 (K8 and K16), thereby altering Sp1-related transcriptional activity, particularly TERT transcription. Telomere dysfunction has been demonstrated to activate DNA damage and affect p53-related cellular senescence [124].

### 3.3.6 Lactylation and aging-related diseases

Studies demonstrate reduced glutamine and accumulated lactate in severely degenerated human nucleus pulposus and aged Sprague-Dawley rat nucleus pulposus, leading to the increase in the lactylation level of cells. Glutamine can reduce AMPKα lactylation by inhibiting glycolysis, prevent disc degeneration, promote autophagy, and inhibit aging of nucleus pulposus cells [125]. The reduced synthesis of collagen by fibroblasts is a key factor in skin aging. Researchers have found that fibroblasts can absorb the extracellular lactate continuously released by poly-L-lactic acid (PLLA) through monocarboxylate transporter 1 (MCT1). This promotes the lactylation of latent transforming growth factor-β binding protein 1 (LTBP1) at lysine 752 via KAT8-dependent regulation and then increases the protein levels of collagen I and collagen III in fibroblasts [126].

Cellular senescence affects the progression of neurodegenerative diseases [8, 127]. Research shows that lactate (as a byproduct of exercise) has potential benefits on various aspects of brain function and disease. Lactate intervention can improve brain-derived neurotrophic factor (BDNF) signaling, angiogenesis signaling (p/t-AKT/ENOS/VEGF), mitochondrial biomarker (SDHA), and metabolic signaling in the hippocampus of aged mice. Lactate may also be used as a therapeutic agent for neurodegenerative diseases in the elderly individuals [128, 129]. EPB41L4A-AS1, a long non-coding RNA (lncRNA) downregulated during aging and in AD, regulate histone acetyltransferase 5-like 2 (GCN5L2) expression to enhance histone acetylation, crotonylation and lactylation near the transcription site of autophagy-related genes, further enhancing astrocyte-mediated clearance of amyloid β peptide (Aβ) and delaying the progression of AD [130]. Some studies also found that lactylation of amyloid precursor protein (APP) decreased in Alzheimer's patients and Alzheimer's model mice and cells. Mass spectrometry analysis of the protein further confirmed that lysine 612 is the key site for APP lactylation, which affects the production and processing of APP amyloid. The lactoyl-mimicking mutant (APP-K612T) can reduce Aβ production and slow cognitive deficits *in vivo* [131].

## 3.4 The Impact of Senescence on lactylation

### 3.4.1 Accumulation of Lactate caused by Metabolic Reprogramming

Senescence is characterized by chronic systemic inflammation and metabolic changes. Research has demonstrated that senescent cells exhibit an augmented tendency for aerobic glycolysis and elevated lactate production, contingent on PDK4, while preserving mitochondrial respiration and redox activity. This phenomenon signifies a distinctive form of metabolic reprogramming with pronounced catabolic characteristics [64]. Comparison of the metabolic status of B cells from young and old donors revealed that senescence was associated with an elevated oxygen consumption rate, particularly an augmented extracellular acidification rate. These rates serve as indicators of OXPHOS and anaerobic glycolysis. Notably, this augmented metabolic state has been observed to correspond to the age-related proliferation of pro-inflammatory B cells. This is accompanied by an increase in lactate secretion levels and the production of autoantibodies following *in vitro* stimulation. *In vitro* studies show that B cells from elderly individuals can induce the polarization of CD4^+^ T cells from young individuals into pro-inflammatory CD4^+^ T cells. This process is facilitated by the lactate-mediated metabolic pathway. This process can be counteracted by targeting lactate-related enzymes and transporters, as well as signaling pathways that support anaerobic glycolysis. Furthermore, lactate induces the production of immune aging B cells with glycolytic characteristics. These cells express the transcripts of various pro-inflammatory molecules and exhibit a heightened metabolic state [132].

### 3.4.2 Lactylation Level in Senescent Cells

Researchers have ascertained that the abundance of histone lactylation decreases significantly during the senescence of skeletal muscle cells in mice. This decline is governed by glycolysis and the regulation of NAD+ content within the organism. It has been postulated that physical exertion, such as running, can enhance histone lactylation levels, thereby restoring the cellular landscape and communication of skeletal muscle in mice. This has the potential to revitalize an organism and enhance its functionality [133]. However, this is in dispute with previous studies, and it may be caused by the research subjects were the fast-twitch fibers of mice [134, 135]. The strategy of using lactate for the treatment of neurodegenerative diseases in the elderly remains controversial [128, 129, 131]. The research results indicate that the increase in lactate levels in the cerebrospinal fluid of elderly individuals can serve as a biological marker of aging, particularly in the brain. It has been determined that the increase in the gene expression ratio of LDH-A to LDH-B is a contributing factor for this phenomenon [136]. Furthermore, a significant increase in lactate levels was observed in aging microglia, the hippocampus, and cerebrospinal fluid of both naturally aging and AD mouse models. This increase in lactate levels results in elevated pan-Kla [66, 137, 138]. However, other studies have demonstrated that nerve excitation can increase lactate and Kla levels in the brain. In additionally, neuronal excitation induced by social frustration stress has been shown to elevate the lactate level and lactylation level in the brain [139].

## 4. Conclusion

Numerous breakthroughs have been achieved in the field of cellular senescence [140-142]. The typical characteristics of senescent cells and the internal and external factors that trigger cellular senescence have been elucidated. The internal factors include elomere attrition, oxidative stress, DNA damage, and mitochondrial dysfunction, while the external factors include radiation, pharmaceuticals, and inflammatory mediators, etc. Recently, the dual roles of cellular senescence in physiological and pathological processes have garnered significant attention, with mounting evidence highlighting its positive contributions to embryonic development and tissue repair [56, 143]. Concurrently, research has delved into its negative implications in promoting the development of aging-related diseases [144, 145].

**Figure 4.**
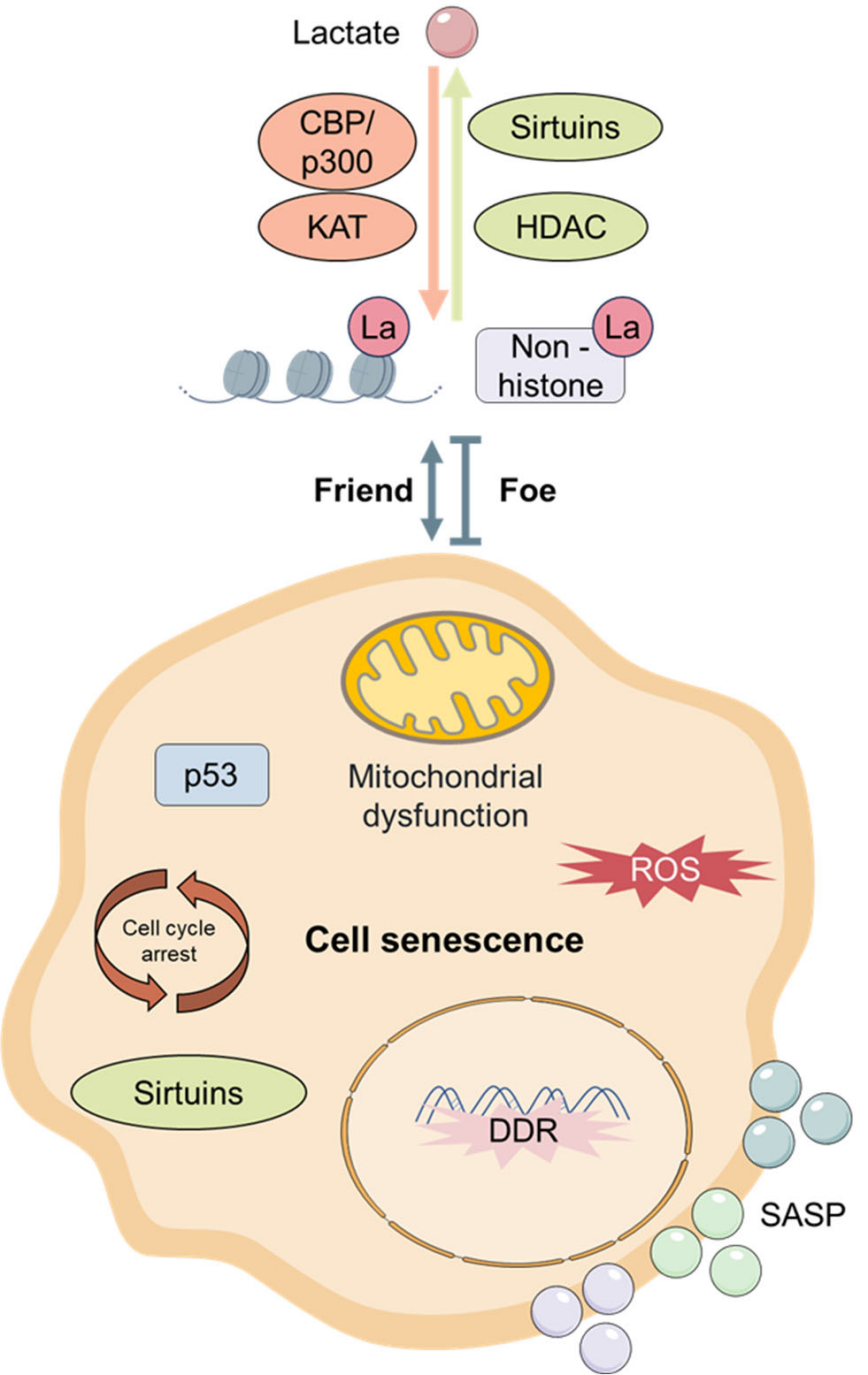
**The regulation between cellular senescence and lactylation**. Lactylation can regulate cellular senescence by modulating the cell cycle, key senescence-related genes, mitochondrial function, and SASP. Meanwhile, cellular senescence can also influence the lactylation level in cells.

The discovery of lactylation modification has opened up a new field in epigenetic research and enriched our understanding of the diversity of protein modifications. Evidently, lactylation is ubiquitous across various cell types, tissues, and organs, playing a pivotal role in regulating key cellular processes and several diseases. In cancer research, lactylation has been observed to promote tumor progression by regulating tumor cell metabolism, facilitating tumor angiogenesis, and enabling tumor cells to evade the immune system. By modulating lactylation levels, it is possible to enhance the efficacy of radiotherapy and chemotherapy and inhibit the proliferation of tumor cells [146-148]. In the field of immunity, lactylation affects the differentiation, activation, and function execution of immune cells, including the regulation of the inflammatory response of immune cells such as T cells and macrophages [149, 150]. In the context of neurodegenerative diseases, lactylation has been shown to be closely related to the survival of neurons and synaptic plasticity. It may be possible to mitigate neuronal damage and dysfunction by modulating lactylation levels, thereby slowing the progression of neurodegenerative diseases [151, 152].

However, research on the close relationship between lactylation levels and cellular senescence is still in its infancy. The existing limited studies have begun to explore the potential association between lactylation levels and cellular senescence. These preliminary studies suggest a direct link between these two phenomena, with lactylation implicated in the process of cellular senescence through diverse mechanisms, including modulation of gene expression, cell cycle regulation, and oxidative stress (Fig. 4). It has been observed that the lactate-related metabolism of senescent cells may increase, and the lactate concentration exerts a substantial influence on the lactylation level of cells. For example, the increased glycolysis level in tumors induces an increase in lactate production, making tumor cells more prone to high-level lactylation. Existing studies also show that lactylation further promotes tumor progression through the regulation of key senescence pathways such as p53 and SASP [89, 124]. Regarding the key "writers" and "erasers" of lactylation, in addition to the regulatory role of lactylation on cellular senescence mentioned above [100, 108], due to the similar enzymes of lactylation and acetylation, numerous phenomena of regulating senescence through acetylation have been reported [105, 153, 154]. Key cellular senescence proteins such as p53 have been identified to have lactylation sites [84, 88], and the impact of these lactylation modification sites on senescence remains to be explored. Consequently, the study of lactylation in cells provides novel insights and potential therapeutic targets for aging-related research. The detection of lactylation levels can serve as a crucial indicator for evaluating cellular senescence and overall health status, providing a basis for the early detection of diseases and personalized treatment.

Since lactylation is an emerging post-translational modification, its specific roles and mechanisms in cellular senescence have not been fully elucidated. At the same time, cellular senescence is a multi-factorial and multi-step process that involves the interaction of various signaling pathways and molecular mechanisms. Although a large number of studies mentioned above have identified lactylation as a key molecule regulating the senescence process, choosing specific targets with therapeutic potential remains a challenge. In addition, due to the similarity of the "writers" and "erasers", lactylation may interact with modifications such as acetylation. Therefore, although a large number of inhibitors targeting the effects of acetylation have been developed [155], the targeting of lactylation regulation is not specific. Small-molecule targeted drugs for lactate regulation have already been reviewed [156], and it is quite challenging to develop drugs or molecular tools that can specifically recognize and target lactylation sites. However, through in-depth research on the regulatory mechanisms and biological functions of lactylation, developing anti-aging strategies targeting lactylation may present a significant opportunity to address the issue of cellular senescence and improve the overall health of the organism.

In summary, lactylation, a pivotal node in the regulatory network of cellular senescence, influences cellular senescence through multifaceted multilevel molecular mechanisms. Conversely, cellular senescence can also affect the accumulation of lactate, leading to changes in cellular function caused by protein lactylation. Although significant breakthroughs have been made in research on lactylation and cellular senescence, there are still many unsolved mysteries, such as the regulatory network, molecular mechanisms, and causal relationships. The relationship between lactylation and cellular senescence is of significant biological importance and clinically relevant in numerous diseases. In the future, with the continuous innovation of technical means and the deepening of interdisciplinary integration, research on the relationship between lactylation and cellular senescence is expected to be transformed into practical intervention strategies, bringing dawn to human health and the prevention and treatment of aging-related diseases.
